# Phylomemetics—Evolutionary Analysis beyond the Gene

**DOI:** 10.1371/journal.pbio.1001069

**Published:** 2011-05-31

**Authors:** Christopher J. Howe, Heather F. Windram

**Affiliations:** Department of Biochemistry, University of Cambridge, Cambridge, United Kingdom

## Abstract

Genes are propagated by error-prone copying, and the resulting variation provides the basis for phylogenetic reconstruction of evolutionary relationships. Horizontal gene transfer may be superimposed on a tree-like evolutionary pattern, with some relationships better depicted as networks. The copying of manuscripts by scribes is very similar to the replication of genes, and phylogenetic inference programs can be used directly for reconstructing the copying history of different versions of a manuscript text. Phylogenetic methods have also been used for some time to analyse the evolution of languages and the development of physical cultural artefacts. These studies can help to answer a range of anthropological questions. We propose the adoption of the term “phylomemetics” for phylogenetic analysis of reproducing non-genetic elements.

Darwin (1809–1882) saw evolution resulting in species being related in a way that could be depicted as a tree. He famously included such a tree as the only figure in *On the Origin of Species by Means of Natural Selection*. However, he was not the first to suggest that species were not immutable, or to depict their relationships in one of a number of possible tree-like ways [1]. Lamarck (1744–1829), for example, had done both of those, and scholars in several other disciplines used trees to represent the relationships among the objects of their study [2]. People studying manuscript texts used the changes incorporated (accidentally or deliberately) when the texts were copied to determine the copying history of extant versions. Those copied from the same earlier version would share variants present in that earlier version, and the copying history was often depicted as a tree. The first recorded example of such a tree (termed a “stemma”—plural stemmata—by manuscript scholars) was probably the one published by Collins and Schlyter in 1827 showing the relationships between a group of medieval Swedish legal texts (reviewed in [2]), and Karl Lachmann (1793–1851) developed principles for the categorisation of errors for this kind of analysis. August Schleicher (1821–1868) published trees of languages from the 1850s onwards. Although there is no evidence that he communicated directly with Darwin, his *Die Darwin'sche Theorie und die Sprachwissenschaft*, published in 1863, referred to *The Origin* as an inspiration, and was addressed to Ernst Haeckel (1834–1919) who worked at Jena, like Schleicher, and was one of the leading proponents of Darwinism in Germany. Schleicher argued that historical linguistic information, such as written texts in Latin, provided a direct demonstration of how languages had developed—something that was not available to the biologist studying the evolution of species. Indeed, the English translation by Bikkers, published in 1869, of his *Darwin'sche Theorie* was called *Darwinism Tested by the Science of Language* [3].

Just over a hundred years after the publication of *The Origin*, in the early 1960s, computer-based methods for reconstructing phylogenetic trees from biological data became available (reviewed in [4]). Numerical taxonomy developed around the same time, and also drew on the increasing availability of computers. Although numerical taxonomy as originally described by Sneath and Sokal did not attempt to draw evolutionary conclusions [5], this followed shortly after [4],[6]. The last few years have seen a major expansion in the application of computer-based phylogenetic methods to the study of texts, languages, and other non-genetic datasets. We will give examples of how the methods are applied to such datasets. We argue that the process of replication with the incorporation of changes is a fundamental one in human cultural activity and beyond. Given the use of the word “meme” to refer to a non-genetic principle that behaves in a genetic way [7], we argue for the adoption of the term “phylomemetics” to refer to the phylogenetic analysis of non-genetic data.

## Phylogenetic Analysis of Manuscripts

**Figure 1:**
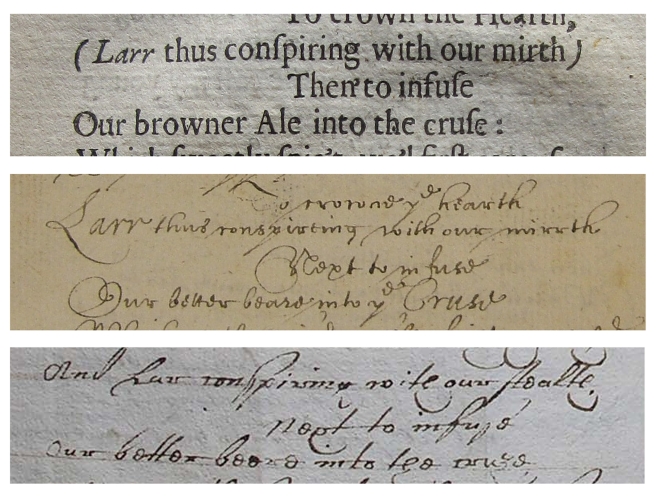
Extracts from the poem “His Age” by Robert Herrick. Figure 2 uses this piece of text as an example of the alignment process. Top panel (Hes in Figure 2) is a printed version from Hesperides, published in 1648 (copy owned by Professor Tom Cain). Middle panel (Ros in Figure 2) is from the Poetical Manuscript Commonplace Book MS 239/23, Rosenbach Museum & Library, Philadelphia. The bottom panel (SJC in Figure 2) is from a verse miscellany, MS S.23, by permission of the Master and Fellows of St John’s College, Cambridge.

**Figure 2:**
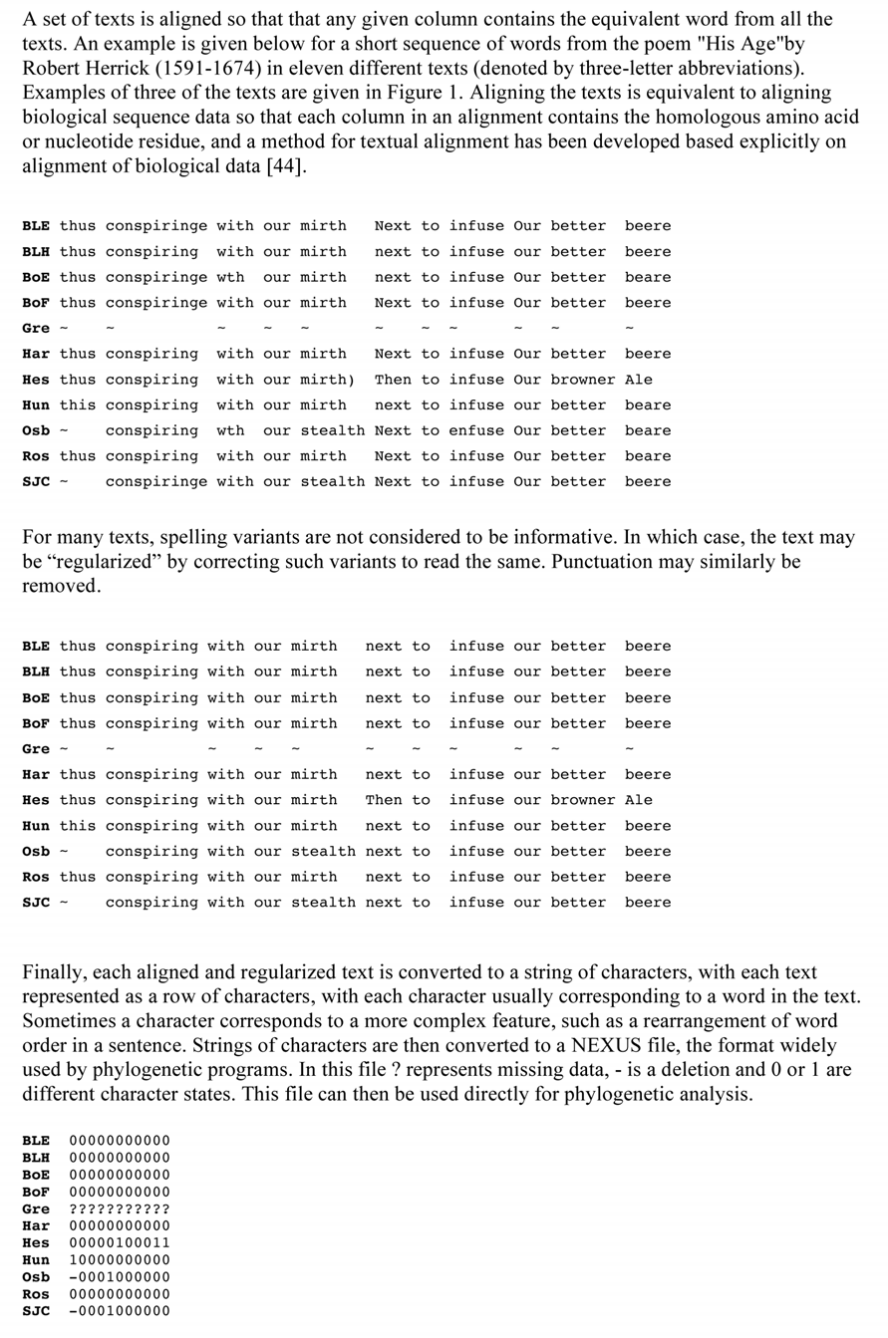
Phylogenetic analysis of texts.

The copying of a manuscript by a scribe with the incorporation of changes that were then propagated when that copy was in turn copied shows clear parallels to the error-prone replication of DNA. Inspired by the development of numerical taxonomy, many scholars started to attempt to apply its methods to questions of classification in the humanities [8]. So, for example, Griffith applied the principles to, among others, the works of Juvenal and Gospel manuscripts [9],[10]. Platnick and Cameron [11] discussed the similarities between cladistics (the basis of parsimony analysis), and the evolution of texts and languages. In the 1980s, Lee applied cladistic software (MacClade and PHYLIP) to St Augustine’s *Quaestiones in Heptateuchum* [12]. Robinson and O'Hara used PAUP in the early 1990s for an analysis of the Old Norse narrative, Svipdagsmal [13]. This demonstrated a very good agreement between a stemma produced by parsimony and one produced by traditional means including, unusually, scribal documentation. The parsimony approach was then applied to parts of Chaucer's *Canterbury Tales* [14] and in 1998, Barbrook et al. used a phylogenetic network method, Split Decomposition, in an analysis of the Prologue to *The Wife of Bath's Tale* [15]. This also showed good agreement between a stemma produced by phylogenetic analysis and one derived by conventional means. The approach for applying phylogenetic methods to texts is simple in principle (Figures 1 and 2). The texts are aligned and then encoded as a string of characters, usually with each character corresponding to a word. The character strings are then used to build a file in exactly the same format as used by phylogenetic tree-building programs, and the file is submitted to the same programs, unaltered. The method has been used to build stemmata for a large number of sets of manuscripts including, in addition to those already mentioned, the Lanseloet van Denemerken story [16], the medieval German legend Parzival [17], parts of the New Testament [18], treatises on the use of the astrolabe [19], writings of St Gregory of Nazianzus [20], historical poems on the Kings of England [21], Dante's *Monarchia* [22], the Mahabharata [23], and the Finnish legend of St. Henry [24]. In general, the conclusions drawn using phylogenetic programs are in agreement with those from conventional scholarship. The method has also been tested using “artificial” traditions, in which volunteers copy a section of text in a predetermined copying history that is then analysed “blind.” Again, the results are generally in agreement with the known copying history [24]–[26].

The use of phylogenetic computer programs in textual analysis has not been without its critics (e.g., [27]). One of the objections often made to the approach is that it does not deal adequately with what scholars call “contamination.” This is where a scribe used more than one copy of a text when making his or her own. Broadly, contamination falls into two varieties. In one, a scribe switched from one copy to another at a particular point. In the other, the scribe used multiple copies simultaneously, to make a patchwork. Contamination has clear parallels in biology, where horizontal gene transfer can result in the incorporation into one organism's genome of a gene from distantly related organisms, or where recombination leads to a sequence that is a hybrid between two parental forms. It is still possible to use phylogenetic analyses with these sets of manuscripts. One approach is to infer trees using subsections of the text and look for individual manuscripts whose position in the tree changes according to the subsection studied [28]. In cases where a scribe switched at a reasonably well-defined point, a method developed by Maynard Smith for mapping recombination sites at the sequence level has been used very successfully for mapping the position in a text where a scribe changed copying source [28]. An alternative approach is to use phylogenetic methods such as Neighbornet or Splitstree, which allow reconstruction of phylogenetic networks. This approach may also be helpful when a scribe used multiple versions simultaneously to make his or her own copy.

Phylogenetic analysis of texts offers scholars a tool for rapid and flexible analysis of texts. Once the primary textual data have been encoded and aligned, it allows scholars to answer in seconds questions such as how the copying history of one chapter compares with another. Its success lies in the fact that copying with incorporation of heritable changes, together with a degree of horizontal transfer, is a reasonable model for the development of manuscripts. But other things evolve in a similar way.

## Phylogenetic Analysis of Languages

Just as 18th century scholars depicted the relationships among languages (as well as the relationships among texts or species) as trees, phylogenetic tree-building programs have also been applied to languages [29],[30]. A widely used approach uses “Swadesh” lists, named after the 20th century linguistic scholar Morris Swadesh, that comprise words with a counterpart in essentially all languages. A set of words is picked from the list and examined in the languages under study. A word that is essentially the same in two languages is counted as conserved. Other words are counted as a substitution. So, for example, “water” in English and “Wasser” in German would be counted as conserved; “eau” in French would be counted as a difference. Datasets built up in this way can then be analysed with the usual phylogenetic inference programs. As well as providing information on tree topology, i.e. which languages form groups to the exclusion of others, these studies often lead to more quantitative conclusions. Just as biological data are sometimes assumed to be evolving in a clock-like fashion, allowing evolutionary divergence times to be estimated, time-calibration of linguistic trees using known divergence times of different languages also allows inferences to be made about, for example, rates of substitution of words [31]. Time calibration of selected points on a tree can also be used to infer dates of important linguistic and anthropological developments, such as the origins of particular languages and timings of population movements [32],[33]. Although some of these inferences with regard to dates are controversial, the same is often true with sequence data [34]. And just as biological data show horizontal gene transfer and texts show contamination, the same is true for linguistic data, which can show “borrowing” or transfer of words between different languages.

## Phylogenetic Analysis of Cultural Artefacts

A number of studies have applied phylogenetic analysis to physical cultural artefacts as well as to languages ([35] and references therein). A challenge here has been to find appropriate ways of coding the features of the artefacts in a way that is appropriate for phylogenetic analysis. Often, an important question has been to determine how well characters can be described by a tree-like evolutionary pattern, or whether other patterns are more appropriate, indicating transfer among different cultural groups. Tëmkin and Eldredge analysed the evolution of two musical instruments, the Baltic psaltery and the cornet [36]. For the Baltic psaltery they included characters such as the presence or absence of a hand-hole, the nature of the ornamentation and the shape of the sound-hole. They recovered a topology that had Slavic and Finnic psalteries as sister groups, with Baltic ones (Latvian and Lithuanian) as a basal group. Given that Slavic and Baltic languages had previously been shown to be sister groups, Tëmkin and Eldredge interpreted this as indicating that the practices underlying instrument building followed geographical rather than linguistic proximity, although the fact that a number of characters showed a distribution that was not congruent with the overall tree indicated examples of convergent evolution or cultural exchange. Analysis of the cornet, by contrast, was much more complex. There was a high degree of reticulation, with fusion of some branches of the tree to form a network, and reconstruction of an unambiguous topology was possible only with the incorporation of historical information. This indicated a large amount of interaction among different instrument builders.

Tehrani and Collard used the degree of reticulation as a measure of cultural contact in elegant analyses of the design and construction of textiles produced in Iran and neighbouring regions [37],[38]. They aimed to test whether these features were passed in a linear way from one generation to the next, or whether there was significant influence, commercial or military, from other sources. They encoded a large number of features, including aspects of the methods used for weaving, and elements of the design such as the use of particular geometric borders, birds, stars, and trees, and assessed the overall quality of fit of the data to a maximum parsimony tree by calculating the retention index (which gives an indication of the number of homoplastic or convergent changes across the tree). They also tested if particular character types (such as technical features of production) gave stronger support for groupings within the tree than other character types (such as motifs in the design). The overall fit to a tree was found to be good, and different character types gave similarly strong support, consistent with the proposal that there was little exchange of these cultural characteristics among tribes.

## General Conclusions

In addition to those described here, there are many other examples of application of phylogenetic analysis to non-genetic data with the aim of recovering evolutionary history. They include studies of written scripts [39] and physical artefacts, such as arrowheads and pottery designs [40],[41], animal behaviour [42], and human organizations and manufacturing structures [43]. In principle, phylogenetic methods can be applied to model the history of any system in which (i) elements can be replicated with the incorporation of changes and (ii) any change between a progeny element and its parent is stably transmitted in subsequent generations. A degree of “horizontal” transfer among elements and/or convergent changes in different lineages may also take place. Horizontal transfer and convergent changes may be recognized by a poor fit between the data and the preferred recovered tree, and can in principle be modelled using network methods of phylogenetic reconstruction. Given the use of the term “meme” to describe reproducing non-genetic elements [7], and units of cultural transmission in particular, we believe the term “phylomemetics” is an appropriate one to refer to phylogenetic analysis of objects other than genes (and their direct products). A search of the web showed occasional uses of this term (e.g., http://papers.ssrn.com/sol3/papers.cfm?abstract_id=1481394), although it did not appear in a search of ISI Web of Knowledge. We believe that it should be formally recognized to refer to this rapidly expanding field.
